# Beneficial Effect of N-Carbamylglutamate in a Neonatal Form of Multiple Acyl-CoA Dehydrogenase Deficiency

**DOI:** 10.1155/2020/1370293

**Published:** 2020-07-14

**Authors:** Sinziana Stanescu, Amaya Belanger-Quintana, Carlos Alcalde Martin, Celia Pérez-Cerdá Silvestre, Begoña Merinero Cortés, Belen Gonzalez Pérez, Carmen Fernández García-Abril, Francisco Arrieta Blanco, Esperanza Palacios Valverde, Mercedes Martínez-Pardo Casanova

**Affiliations:** ^1^Unidad de Enfermedades Metabólicas, Hospital Ramón y Cajal, IRYCIS, Crta de Colmenar Viejo, Km 9,100, PC 28034, Madrid, Spain; ^2^Servicio de Pediatria, Hospital Universitario Rio Hortega, C/Dulzaina 2, PC 47012, Valladolid, Spain; ^3^Centro de Diagnóstico de Enfermedades Moleculares, Centro de Biología Molecular, Universidad Autónoma de Madrid, CIBERER, IdiPAZ, C/ Francisco Tomás y Valiente 7, PC 28049, Madrid, Spain; ^4^Unidad de Cuidados Intensivos Pediátricos y Neonatales, Hospital Clínico Universitario, Av. Ramón y Cajal 3, PC 47003, Valladolid, Spain

## Abstract

*Background*. Multiple acyl-CoA dehydrogenase deficiency is an autosomal recessive disorder of the amino acid metabolism and fatty acid oxidation due to the deficiency of the electron transfer protein or electron transfer protein ubiquinone oxidoreductase. The clinical picture ranges from a severe neonatal lethal presentation to late myopathic forms responsive to riboflavin. Up to now, there is no effective treatment for the neonatal form, which exhibits severe metabolic acidosis, hyperammonemia, hypoketotic hypoglycemia, and rhabdomyolysis. We present the case of a child who has had a good long-term outcome after a typical neonatal onset, with a dramatic drop in ammonia levels during the initial metabolic decompensation crisis and adequate control even during intercurrent diseases thereafter with N-carbamylglutamate treatment.

## 1. Introduction

Multiple acyl-CoA dehydrogenase deficiency (MADD; MIM 231680), also known as glutaric aciduria type II, is an autosomal recessive disorder affecting the fatty acid and amino acid metabolism. Most of the cases are due to mutations in the genes and secondary enzymatic deficiency of either the electron transfer flavoprotein (ETF) or electron transfer flavoprotein ubiquinone oxidoreductase (ETFDH) [1, 2]. In some patients, MADD-like disorders may be due to mutations in riboflavin transporter genes (SLC52A1 [MIM: 607883], SLC52A2 [MIM: 607882], and SLC52A3 [MIM: 613350]) [3], as well as in the mitochondrial FAD transporter gene (SLC25A32 [MIM: 610815]) [4].

The clinical presentations of MADD are heterogeneous [5]. The most common and best known is the mild myopathic late onset form, which manifests in late infancy or adolescence with myopathy or hypoglycemic encephalopathy and usually responds to high doses of riboflavin supplementation [6–8]. Children with a neonatal debut have severe metabolic acidosis, hyperammonemia, hypoketotic hypoglycemia, encephalopathy, and Reye-like syndrome; these infants may also have congenital malformations such as polycystic kidney and can develop an early form of cardiomyopathy [1, 2]. No treatment has been found effective for the neonatal form, which frequently has a lethal outcome.

As in other inborn errors of metabolism, high ammonia levels are an important sign of metabolic decompensation in the neonatal form of MADD. The levels and duration of hyperammonemia correlate with the survival and neurological outcome of the patient and therefore require an early and aggressive treatment. The reason for high ammonia levels in MADD is not completely understood. It seems related with the competitive inhibition of N-acetylglutamate synthetase by certain organic acids, leading to reduced N-acetylglutamate synthesis and secondary impairment of the urea cycle [9]. N-Carbamylglutamate (NCG) or carglumic acid is a structural analog of N-acetylglutamate, and it appears to be useful in the treatment of hyperammonemia in other organic acidemias, such as propionic, methylmalonic, or isovaleric acidemia [10–13].

We present the case of a child with a neonatal debut of MADD with a good long-term outcome and successful treatment with NCG both during the initial metabolic decompensation and later intercurrent diseases. Consent for publication has been obtained from the parents.

## 2. Case Report

**Figure 1:**
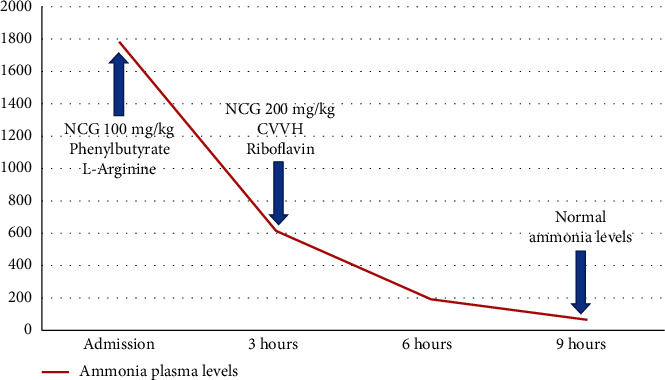
Evolution of ammonia levels (*μ*mol/L) during the neonatal decompensation.

A full-term female infant was born via vaginal delivery from nonconsanguineous parents; the pregnancy was complicated by maternal hypothyroidism. She had a birth weight of 2.9 kg and a normal perinatal period. Neonatal screening was not expanded in her area. On the 8^th^ day of life, she was brought to the hospital because of vomiting, a low conscience level, and hypotonia in the previous hours. Urgent blood determinations revealed hypoketotic hypoglycemia (glycemia: 0.9 mmol/L and ketone bodies: 0.4 mmol/L), metabolic acidosis (pH: 7.05, excess bases: −26 mmol/L, and lactate: 2.9 mmol/L), and severe hyperammonemia (1744 *μ*mol/L, normal values (NV) for neonates < 110 *μ*mol/L). Laboratory examinations also showed rhabdomyolysis (CK: 2379 U/L and NV: 38–174 U/L), high transaminase level (AST: 117 U/L, NV: 4–50 U/L; ALT: 160 U/L, NV: 5–40 U/L), and coagulopathy. After the initial measures (intubation, correction of hypoglycemia, and saline volume expansion), a first dose of NCG (100 mg/kg/dose) was given by the nasogastric tube. The patient was then transferred to a pediatric intensive care unit, and continuous venovenous hemodiafiltration (CVVH) was started 4 hours after admission. With the suspicion of a metabolic derangement, contact with a metabolic reference center was established, and she was given cofactor treatment with riboflavin, cobalamin, biotin, thiamine, and carnitine. The levels of ammonia dropped dramatically after the first dose of NCG and normalized completely within the first 9 hours of treatment (Figure 1). The rest of the biochemical abnormalities also normalized in the first days after admission.

Two days after the initial debut, the results of the urine gas chromatography were received. They showed increased excretion of glutaric acid (9290 mmol/mol creatinine; NV: <5), 2-hydroxyglutaric acid (290 mmol/mol creatinine; NV: <62), acylglycines (hexanoylglycine: 32 mmol/mol creatinine, NV: not detectable; suberylglycine: 16 mmol/mol creatinine, NV: not detectable), and dicarboxylic acids; the plasma samples revealed elevation of C4–C18 acylcarnitines and proline (318 *µ*mol/L, NV: <270), suggesting the diagnosis of MADD. Later, mutational analyses of the ETF/ETFDH genes confirmed that the patient is a compound heterozygous in the ETFDH gene. Previous reported mutations were found: c.24 + 4T > C p.Ser82Pro, in exon 3 [14] and c.34+5G > C, affecting the donor splice site of intron 1 [15]. In in silico evaluation, using Saphetor software (https://saphetor.com/), c.34+5G > C mutation was classified as likely pathogenic (type 2 variant), and c.244T > C was classified as pathogenic mutation (type 1 variant).

Once the diagnosis was established, riboflavin (300 mg/day) and carnitine treatment was maintained, and coenzyme Q10 supplementation was added [16–18]. Considering the excellent response of hyperammonemia to NCG, we maintained it as a chronic treatment (100 mg/kg/day). The tablets for oral suspension (200 mg) were mixed in water (5 ml for each tablet) taken initially by the nasogastric tube and later orally. Enteral feeding begun in the first few days with a diet consisting of natural protein restriction (0.7–1 g/kg/day) supplemented with a leucine-free amino acid formula, accounting for a total amount of protein of 2–2.5 g/kg/day. Dietary management also included an adequate energy supply (caloric intake 100–120 kcal/kg/day), combined with avoidance of prolonged fasting (meals every 3-4 hours during the first year of life and every 5-6 hours since then). The parents have received nutritional education and support in every hospital visit, which has been scheduled every month in the first year of life and every 3 months thereafter.

The child is now 4 years old and has had an excellent outcome. She has a very slight language learning disability and no signs of cardiomyopathy. Anthropometrical measurements are normal according to the WHO standards: p50–75 for length and weight and p25 for head circumference.

So far, she has had only two mild metabolic decompensations coinciding with respiratory infections. Discrete elevations of hepatic transaminases and creatine kinase (maximum 1200 U/L) were detected during these episodes, but no acidosis or hyperammonemia. As a preventive measure, the dose of NCG was doubled during these episodes.

## 3. Discussion

To our knowledge, there are no previous reports of good outcome in the neonatal form of MADD. Several reasons could explain the difference between our patient and previous experience. One important factor is the speed in which the diagnosis and treatment of both hyperammonemia and MADD were made. Probably, the good neurologic outcome of our patient is due to the dramatically rapid normalization of the ammonia levels and the lack of further severe decompensation crisis.

High ammonia levels are the cause of mortality and neurological sequelae in several metabolic diseases. The mechanism of hyperammonemia in MADD is not fully understood but may be similar to the one involved in other organic acidemias such as propionic, methylmalonic, or isovaleric acidemia. In these cases, high ammonia levels are due to a competitive inhibition of the NAGS enzyme by various organic acids which causes a secondary dysfunction of the urea cycle [9–13]. MADD patients also have a high excretion of various organic acids, including isovaleric or dicarboxylic acids that may have a similar inhibitory effect on NAGS activity [9, 12]. Nevertheless, there are no studies to prove this hypothesis.

The use of NCG for the treatment of hyperammonemia in neonatal MADD forms has not been reported previously. This treatment, together with the rapid initiation of hemodialysis, allowed for a rapid normalization of ammonia levels. Considering the good initial response to NCG and the uncertainty of the evolution of this patient in the first months of life, we decided to maintain this treatment in the long term. To this date, at the age of 4 years, the patient has not presented any new episodes of hyperammonemia, and decompensation crisis has been mild. We can speculate that NCG has a “protective” effect, maintaining a pool of substrate for the NAGS enzyme and activating the urea cycle, which prevents hyperammonemia.

Consistent with the EMA label, the recommended daily dose of NCG ranges from 10 to 100 mg/kg/day divided into two to four doses. In coma due to hyperammonemia, a dose up to 250 mg/kg/day may be necessary. NCG is for oral use only (ingestion or via a nasogastric tube using a syringe, if necessary). The tolerance is generally good; only a few side effects have been so far reported including elevated transaminase levels or sweating.

Recently, there is an increased interest for the chronic treatment with NGG in patients with organic acidemias who present frequent episodes of hyperammonemia [11]. Although the medical literature is sparse and there are no comparative studies, the cases presented so far seem to show a reduction of the hyperammonemia episodes. Nevertheless, there are no clinical guidelines for the chronic treatment with NCG in patients with organic acidemias.

Another reason for the good outcome of our patient might be the diet established, which included measures to compensate for both amino acid and fatty acid routes affected by the disease. Also, we cannot completely rule out a response to riboflavin even if the patient has a neonatal severe form.

In conclusion, our experience suggests that a leucine-restrictive diet, riboflavin, coenzyme Q10 supplementation, and carglumic acid might be considered for the treatment of patients with a neonatal presentation of MADD. The use of NCG, in particular, seems useful to control hyperammonemia both during an acute crisis and in the long term, although further trials are needed to demonstrate its beneficial effect.

## Data Availability

The data used to support the findings of this study are available from the corresponding author upon request.

## Abbreviations

MADD: Multiple acyl-CoA dehydrogenase deficiency
ETF: Electron transfer flavoprotein
ETFDH: Electron transfer flavoprotein ubiquinone oxidoreductase
NCG: N-Carbamylglutamate
CVVH: Continuous venovenous hemofiltration.
